# Distribution and Risk Factors of Scrub Typhus in South Korea, From 2013 to 2019: Bayesian Spatiotemporal Analysis

**DOI:** 10.2196/68437

**Published:** 2025-09-10

**Authors:** Jeehyun Kim, Penelope Vounatsou, Byung Chul Chun

**Affiliations:** 1 Department of Preventive Medicine, College of Medicine, Korea University, 73 Goryeodae-ro, Seoungbuk-gu, Seoul, 02841, Republic of Korea, 82 2-2286-1169; 2 Swiss Tropical and Public Health Institute, University of Basel, Basel, Switzerland

**Keywords:** Orientia tsutsugamushi, vector-borne diseases, Rodentia, spatiotemporal analysis, One Health

## Abstract

**Background:**

Scrub typhus (ST), also known as tsutsugamushi disease, is a common febrile vector-borne illness in South Korea, transmitted by trombiculid mites infected with *Orientia tsutsugamushi*, with rodents serving as the main hosts. Although vector-borne diseases like ST require both a One Health approach and a spatiotemporal perspective to fully understand their complex dynamics, previous studies have often lacked integrated analyses that simultaneously address disease dynamics, vectors, and environmental shifts.

**Objective:**

We aimed to explore spatiotemporal trends, high-risk areas, and risk factors of ST by simultaneously incorporating host and environmental information.

**Methods:**

ST cases were extracted from the 2013‐2019 Korea National Health Insurance Service data at 250 municipal levels and by epidemiological weeks (*International Classification of Diseases, Tenth Revision, Clinical Modification code*: A75.3). Data on potential risk factors, including the maximum probability of rodent presence, area of dry field farming, forest coverage, woman farmer population, and financial independence, were obtained from publicly available sources. In particular, the maximum rodent presence probability was estimated using a maximum entropy model incorporating ecological and climate variables. Spatial autocorrelation was assessed using Global Moran *I* statistics with 999 Monte Carlo permutations. Spatial and temporal clusters were identified using Getis-Ord $G_{i}^{*}$ and hot and cold spot trend analyses. Bayesian hurdle models with a spatiotemporal interaction term, accounting for zero-inflated Poisson distribution, were used to identify associations between ST incidence and regional factors. Stratification analyses by gender and age group (0‐39, 40‐59, 60‐79, and ≥80 years) were performed.

**Results:**

Between 2013 and 2019, 95,601 ST patients were reported. ST incidence had positive spatial autocorrelation (*I*=0.600; *P*=.01), with spatial expansion from southwestern to northeastern regions. Spatiotemporal models demonstrated better fit compared with spatial and temporal models, as indicated by lower Watanabe-Akaike information criterion (WAIC) values. Municipalities with higher rodent suitability (β coefficient=0.618; 95% credible interval [CrI] 0.425‐0.812) and lower financial independence from central government (β coefficient=−0.304; 95% CrI −0.445 to −0.163) had higher likelihoods of increased ST incidence, even after adjusting for spatiotemporal autocorrelation. However, risk factors varied by age group: among individuals aged 40 years or older, ST incidence was positively associated with rodent suitability, while patients in the 0‐39 years age group showed no association with rodent suitability (β coefficient=0.028; 95% CrI −0.072 to 0.126), and ST incidence was negatively associated with the women farmer population (β coefficient=-0.115; 95% Crl=−0.223 to −0.006).

**Conclusions:**

This is the first study to investigate ST in South Korea using a spatiotemporal framework grounded in a holistic One Health perspective. We elucidated the critical role of spatiotemporal dynamics in ST distribution, highlighting rodent suitability and economic independence as key drivers of disease distribution. Our findings lay the groundwork for evidence-based, region-specific intervention strategies and may inform targeted public health strategies in South Korea and other settings with similar ecological conditions.

## Introduction

Scrub typhus (ST), also known as tsutsugamushi disease, is a vector-borne illness transmitted by trombiculid mites infected with *Orientia tsutsugamushi* [1]. It is a common cause of acute febrile illness across the Asia-Pacific region, including South Korea [1]. Clinical manifestations range from acute fever, headache, rash, and skin eschars at mite bite sites to severe conditions such as acute respiratory distress syndrome, kidney failure, and multiorgan failure, particularly in the absence of timely antibiotic treatment [1,2]. Despite advances in diagnostics and treatment for decades, no effective vaccine exists [2]. The disease primarily occurs during the chigger mite breeding season, typically with an autumn peak in South Korea [3], a seasonal pattern also observed in leptospirosis and hemorrhagic fever with renal syndrome, with rodents as the primary host [4,5].

Analyzing ST requires a holistic approach that considers individual attributes, vectors, and environmental factors, aligned with the One Health perspective [6]. The One Health perspective can improve our understanding of the disease and help identify risk factors by addressing various factors simultaneously, enabling more effective prevention and interventions in person, time, and place [7,8]. In South Korea, *Apodemus agrarius*, the most common rodent species, has been identified as an important reservoir of zoonotic pathogens, including *Orienta tsutsugamushi*, highlighting the need to integrate host surveillance within a One Health framework to support comprehensive public health strategies [9]. However, previous studies have limitations in simultaneously addressing various factors. Most surveys focused primarily on individual experiences [10,11], and ecological studies have addressed environmental factors without considering the distribution of vectors and rodents [12-15]. Previous studies exploring the association between vector distribution and ST infections have overlooked the information environment and human behavior [16], such that they had limitations in suggesting robust risk factors.

Studies on vector-borne diseases have mandated integrated spatiotemporal analyses that encompass disease dynamics, vectors, and environmental shifts. Spatiotemporal methods can help identify high-risk areas [17,18], understand disease transmission [19], monitor disease trends [19], and assess the impact of environmental factors [17,19]. Although previous studies considered both the spatial and temporal axes simultaneously, the studies had limitations in pursuing the One Health approach [12,13,20].

Therefore, we aimed to investigate the spatiotemporal trends, high-risk areas, and risk factors of ST by integrating hosts and environmental information, and spatiotemporal dynamics. These findings are expected to provide a robust foundation for evidence-based policies to alleviate the disease burden, with particular emphasis on rodent distribution as a central determinant of ST risk.

## Methods

### Data

#### Scrub Typhus Data

Data on ST cases from 2013 to 2019 were extracted from the National Health Insurance Service (NHIS) of South Korea. We used the NHIS claims data, which cover the entire Korean population under the country’s universal health insurance system. Unlike data from the national infectious disease surveillance system, which may be affected by periodic changes in reporting criteria, NHIS data are stable and structured, making them suitable for long-term trend analysis [21]. Therefore, although surveillance reporting criteria were revised in 2019, their impact on NHIS data were limited, and we retained the study period to 2013‐2019 to ensure sufficient temporal coverage.

ST was defined according to the *International Classification of Diseases, Tenth Revision*, Clinical Modification code (ICD-10-CM code) of A75.3. The data were extracted in 250 municipal-level administrative boundaries on the spatial axis and were extracted in epidemiological week, commonly referred to as Morbidity and Mortality Weekly Report weeks [22], and calendar month on temporal axis. In South Korea, administrative divisions are organized into 4 levels: National, State (Sido), Municipal (Si [cities], Gun [counties], Gu [districts]), Town (Eup-Myun-Dong). The data included information on residential address coded by municipality, gender (men and women), and age group, which were extracted in 20-year interval category (0‐39 years, 40‐59 years, 60‐79 years, and ≥80 years of age) because of privacy-related data extraction regulations of the data provider. Patients with complete data were included in the analyses.

As the standard analysis unit, municipal-level administrative boundaries in 2019 and epidemiological weeks were used to ensure comparability across years. For spatial analysis, a municipal-level shapefile was obtained from the publicly accessible Statistical Geographic Information Service [23]. Population data to calculate incidence per 100,000 population for descriptive analysis was extracted from Statistics Korea [24].

#### Potential Risk Factors

The maximum probability of rodent presence by municipality was selected as a potential risk factor of ST to consider reservoir distribution, aligned with the One Health perspective [6]. Based on previous studies, information on the maximum probability of the presence of rodents was made using the maximum entropy model (MaxEnt) [25], with point data of rodent existence, human population density [26], and various environmental raster data: 2014‐2018 average enhanced vegetation index (EVI) [27], land surface temperature (LST) data [27], and a digital elevation model (DEM) [27]. Rodent existence point data were extracted from the 2014‐2018 National Ecosystem Survey data from the Ministry of Environment [28] regardless of the rodent species and deleted the redundant points. The raster data of human population density in 1×1 km resolution was extracted from “The spatial distribution of population density in 2018, South Korea” in WorldPop hub [29]. The raster data of EVI and LST were extracted from moderate-resolution imaging spectroradiometer (MODIS) remote sensing data using *MODIStsp* package [30] in R software (version 4.2.1 R Foundation for Statistical Computing). Monthly EVI raster at 1×1 km resolution and daily daytime and nighttime LST at 1×1 km were averaged. The DEM raster at 90×90 m was extracted from the National Spatial Data Infrastructure Portal of the Ministry of Land, Infrastructure, and Transport [31]. The study area of MaxEnt was set outside of a 10 km buffer from rodent existence points for the model to study various environmental areas beyond the point where rodents were discovered. The results of the MaxEnt model showed a better prediction than the random model, with an area under the curve of 0.690. The maximum rodent suitability by municipality was then extracted from the results of the MaxEnt model.

Besides maximum rodent suitability, area of dry field farming [10,11,32,33], forest coverage [32], woman farmer population [11,33], and financial independence as a socio-economic status of the municipality [34] were included based on previous studies and relevance to ST transmission (Table 1). The woman farmer population was defined as the number of females formally engaged in farming activities per municipality and year and used as an ecological indicator of agricultural exposure of women. This variable was selected based on the higher burden of ST among women in Korea and previous findings suggesting strong associations between agricultural activity and ST incidence in women [11,33]. Information on each variable was extracted in year and municipality level. In addition, the population of women and men farmers was extracted from Korean statistical information service at the year and state level to compare the gender ratio in farming population by year and state as supplementary information. Although other potential predictors such as meteorological factors were considered, relevant environmental conditions were already incorporated into rodent suitability construction. Variables were selected considering epidemiological plausibility, municipal-level data availability, and efforts to reduce collinearity among predictors. The shapefile was obtained from the National Spatial Data Infrastructure Portal [31].

**Table 1.** Description and data sources of outcome and potential risk factors for scrub typhus incidence in South Korea, 2013-2019.

Variables	Description	Sources
Scrub typhus		
Cases (n)	Cumulative scrub typhus cases by municipality and epidemiological week	NHIS^a^
Potential risk factors		
Maximum rodent suitability	Maximum possibility of rodent presence made by maximum entropy model using point data of rodent existence and various environmental raster data, per municipality (range: 0.0‐1.0)	NESD^b^, WorldPop hub, MODIS^c^, NSDIP^d^
Financial independence (%)	Total earned income over total expense, per municipality and year	KOSIS^e^
Forest area (*m*^2^)	Land that forms forests and fields, per municipality and year	KOSIS^e^
Dry field farming area (*m*^2^)	Land that mainly grow plants that do not apply water, per municipality and year	KOSIS^e^
Woman farmer population (n)	Population of women farmers, per municipality and year	KOSIS^e^
Offset		
Population (n)	Human population based on the resident registry in, per municipality	KOSIS^e^

a NHIS: National Health Insurance Service.

b NESD: National ecosystem survey data.

c MODIS: Moderate resolution imaging spectroradiometer.

d NSDIP: National Spatial Data Infrastructure Portal.

e KOSIS: Korean statistical information service.

### Statistical Analysis

#### Descriptive Analysis

The frequency and incidence of ST were analyzed with respect to calendar year, gender, and age group, similar to the approach used in a previous study [21]. Descriptive statistics, including mean, SD, minimum, median, first quartile, third quartile, maximum, and coefficient of variation, were used to summarize the baseline information for the variables. As the dataset overlaps with that in the previous study [21], relevant findings were referenced. However, this study introduces spatiotemporal analysis methods and adopts more suitable age groupings, offering a refined understanding of ST dynamics over time and space.

To examine the spatiotemporal distribution of ST incidence, choropleth maps depicting ST incidence per 100,000 population with quintile intervals were created, stratified by calendar year, gender, and age group. Since maps only visualize data and cannot provide definitive conclusions due to various sources of uncertainty, Global Moran’s *I* statistics were used with 999 Monte Carlo permutations (*P*<.05) to quantify the annual spatial autocorrelation in ST incidence. The Moran’s *I* index ranges from −1 to 1, with a positive value signifying a positive spatial autocorrelation. This implies that regions with high variable values are likely to be close to each other. The significant results of Global Moran’s *I* statistics could provide verification to consider spatial autocorrelation for further analysis. Subgroup analyses were conducted based on gender and age group.

Furthermore, a Getis-Ord $G_{i}^{*}$ analysis stratified by calendar year, gender, and age group was performed to identify municipal-level clusters within each stratum. Municipalities with high positive *z* scores, resulting from the Getis-Ord $G_{i}^{*}$ analysis, indicated “hot spots,” signifying spatial clustering of high values. Conversely, “cold spots” were characterized by low negative *z* scores, indicating spatial clustering of low values surrounded by other low-value municipalities. The results of Getis-Ord $G_{i}^{*}$ analysis provide information on locations with either high or low value of ST incidence that are statistically significantly clustered.

The hot and cold spot trend (HCT) analysis was performed, which is the visualization of a space-time cube in 2 dimensions. The HCT depicts increasing and decreasing *z* scores resulting from Mann–Kendall statistics, classified as “up trend,” “down trend,” or “no significant trend” with confidence levels of 90%, 95%, and 99%, respectively. Thus, while Getis-Ord $G_{i}^{*}$ analysis tests clustering on spatial axis, HCT analysis examines statistical trends across both spatial and temporal axes. The results could test whether the ST clusters expand in spatial and temporal perspectives. The three-dimensional space-time cube (x, y, and t) was generated using the “Create Space Time Cube From Defined Locations” tool, with 250 municipal polygons as spatial units and one calendar month as temporal units, resulting in 21,000 bins (250 spatial units×84 temporal units). Missing values in each bin were replaced with zeros. Spatial analyses, including mapping, assessment of spatial autocorrelation, and cluster identification, were performed using R version 4.2.1 [35], and HCT analysis was conducted using ArcGIS Pro version 3.3.1 (Esri) [36].

#### Modeling

Modeling was performed to explore the association between ST incidence and each potential risk factor after adjusting the effect of space and time trend. For modeling, continuous predictors, including rodent suitability, were standardized due to varying units. Age-standardized ST incidence per 100,000 population was summarized using population data from Population and Housing Census in 2015 [37]. Univariable linear regression was conducted for each variable using age-standardized ST incidence as dependent variable. Variables with a *P* value ≥.25 [38] were identified for exclusion in the multivariable regression (Table S1 in Multimedia Appendix 1). Multicollinearity was assessed using a variance inflation factors threshold of 10 in the multivariable regression (Table S2 in Multimedia Appendix 1).

The primary analytical method used was the Bayesian hurdle Poisson spatiotemporal analysis using the Integrated Nested Laplace Approximation (INLA) approach [39,40], a computationally efficient alternative to the frequently used Markov Chain Monte Carlo simulations [40]. INLA delivers precise and fast results for latent Gaussian models [40] and is widely applied in spatiotemporal models to handle model complexity and vast database dimensions [41,42].

The zero-inflated Poisson distribution was managed using the hurdle Poisson model due to the excessive zeroes in the ST incidence data [39,43]. A hurdle model outperforms a zero-inflated model when covariates have the possibility of zero deflation when using excess zero data [43]. Thus, a hurdle model was constructed with 2 likelihoods (Bernoulli and truncated Poisson) in the INLA [39,44]. The Bernoulli distribution, implemented as “binomial” in the R-INLA, modeled the probability of any case occurrence [40,44], while the zero-truncated Poisson distribution, specified as “poisson,” captured the incidence when cases occurred [44]. The age-standardized incidence of ST per municipality, $i\left(i=1,\ldots ,250\right)$ and epiweek, $t\left(t=1,\ldots ,365\right)$ can be represented as $Y_{it}$, using a hurdle Poisson model as follows:


$$P\left(Y_{it}=y|p,\mu \right)=\left\{ \begin{array}{l} p\;\;\;\;\;\;\;\;\;\;\;\;\;\;\;\;\;\;\;\;\;\;\;\;\;\;\;\;\;\;\;\;\;\;\;\;\;y_{it}=0,\\ \left(1-p\right)\frac{e^{-\mu }\mu^{y}/y!}{1-e^{-\mu }}\;\;\;\;\;\;\;y_{it}>0 \end{array}\right.$$

where p denotes the probability that a participant belongs to the zero component. First, to examine the necessity of spatiotemporal interaction term compared to each spatial and temporal term, three base models were constructed as follows:


$$Spatial\;model\;0\;\left\{ \begin{array}{l} logit\left(p_{it}\right)=\;\beta_{0}^{p}+u_{i}^{p}+v_{i}^{p}\\ \log\left(\mu_{it}\right)=\;\beta_{0}^{\mu }+u_{i}^{\mu }+v_{i}^{\mu } \end{array}\right.$$

$$Temporal\;model\;0\;\left\{ \begin{array}{l} logit\left(p_{it}\right)=\;\beta_{0}^{p}+\gamma_{t\;}^{p}+\phi_{t}^{p}\\ \log\left(\mu_{it}\right)=\;\beta_{0}^{\mu }+\gamma_{t}^{\mu }+\phi_{t}^{\mu } \end{array}\right.$$

$$Spatiotemporal\;model\;0\left\{ \begin{array}{l} logit\left(p_{it}\right)=\;\beta_{0}^{p}+u_{i}^{p}+v_{i}^{p}+\gamma_{t\;}^{p}+\phi_{t}^{p}+\delta_{it}^{p}\\ \log\left(\mu_{it}\right)=\;\beta_{0}^{\mu }+u_{i}^{\mu }+v_{i}^{\mu }+\gamma_{t}^{\mu }+\phi_{t}^{\mu }+\delta_{it}^{\mu } \end{array}\right.$$

where $\mu_{it}$ denotes the exponential value of ST incidence for municipality i and epiweek t, $\beta_{0}$ denotes intercept [45]. The model contains $u_{i}$ as an intrinsic conditional auto-regressive (iCAR) term, $v_{i}$ as a spatially unstructured term, $\gamma_{t}$ as a temporally structured effect using an autoregressive model of order 1, and $\phi_{t}$ that has been specified as $\phi_{t}\sim Normal\left(0,\frac{1}{\tau_{\phi }}\right)$ [46]. The term $\delta_{it}$ is an interaction term of spatial and temporal structured effect [46]. The spatial weight matrix, which determines the iCAR term, represents a neighboring relationship among the 250 municipalities. The neighbor matrix was constructed using k-nearest neighborhood, with k as 3. Priors followed the default options of R-INLA.

In addition, to assess the effect of potential risk factors other than maximum rodent suitability on ST incidence, a term for these factors was added to each model. When $\beta_{j}$ denotes regression parameters of each potential risk factor excluding the maximum possibility of rodent presence, the model was constructed as follows:


$$Spatial\;model\;1\left\{ \begin{array}{l} logit\left(p_{it}\right)=\;\beta_{0}^{p}+\sum_{j}\beta_{j}^{p}\times Potential\;risk\;factors_{it,j}^{p}+\\ u_{i}^{p}+v_{i}^{p}\\ \log\left(\mu_{it}\right)=\;\beta_{0}^{\mu }+\sum_{j}\beta_{j}^{\mu }\times Potential\;risk\;factors_{it,j}^{\mu }+\\ u_{i}^{\mu }+v_{i}^{\mu } \end{array}\right.$$

$$Temporal\;model\;1\left\{ \begin{array}{l} logit\left(p_{it}\right)=\;\beta_{0}^{p}+\sum_{j}\beta_{j}^{p}\times Potential\;risk\;factors_{it,j}^{p}+\\ \gamma_{t\;}^{p}+\phi_{t}^{p}\\ \log\left(\mu_{it}\right)=\;\beta_{0}^{\mu }+\sum_{j}\beta_{j}^{\mu }\times Potential\;risk\;factors_{it,j}^{\mu }+\\ \gamma_{t}^{\mu }+\phi_{t}^{\mu } \end{array}\right.$$

$$Spatiotemporal\;model\;1\left\{ \begin{array}{l} logit\left(p_{it}\right)=\;\beta_{0}^{p}+\sum_{j}\beta_{j}^{p}\times Potential\;risk\;factors_{it,j}^{p}+\\ u_{i}^{p}+v_{i}^{p}+\gamma_{t\;}^{p}+\phi_{t}^{p}+\delta_{it}^{p}\\ \log\left(\mu_{it}\right)=\;\beta_{0}^{\mu }+\sum_{j}\beta_{j}^{\mu }\times Potential\;risk\;factors_{it,j}^{\mu }+\\ u_{i}^{\mu }+v_{i}^{\mu }+\gamma_{t}^{\mu }+\phi_{t}^{\mu }+\delta_{it}^{\mu } \end{array}\right.$$

Finally, to assess the risk of rodent suitability on ST incidence after accounting for other potential risk factors, and vice versa, a term of rodent suitability was added to each model, where κ denotes the coefficient for rodent suitability, as follows:


$$Spatial\;model\;2\left\{ \begin{array}{l} logit\left(p_{it}\right)=\;\beta_{0}^{p}+\sum_{j}\beta_{j}^{p}\times Potential\;risk\;factors_{it,j}^{p}+\\ \kappa^{p}\times Rodent\;suitability_{it}^{p}+u_{i}^{p}+v_{i}^{p}\\ \log\left(\mu_{it}\right)=\;\beta_{0}^{\mu }+\sum_{j}\beta_{j}^{\mu }\times Potential\;risk\;factors_{it,j}^{\mu }+\\ \kappa^{\mu }\times Rodent\;suitability_{it}^{\mu }+u_{i}^{\mu }+v_{i}^{\mu } \end{array}\right.$$

$$Temporal\;model\;2\left\{ \begin{array}{l} logit\left(p_{it}\right)=\;\beta_{0}^{p}+\sum_{j}\beta_{j}^{p}\times Potential\;risk\;factors_{it,j}^{p}+\\ \kappa^{p}\times Rodent\;suitability_{it}^{p}+\gamma_{t\;}^{p}+\phi_{t}^{p}\\ \log\left(\mu_{it}\right)=\;\beta_{0}^{\mu }+\sum_{j}\beta_{j}^{\mu }\times Potential\;risk\;factors_{it,j}^{\mu }+\\ \kappa^{\mu }\times Rodent\;suitability_{it}^{\mu }+\gamma_{t}^{\mu }+\phi_{t}^{\mu } \end{array}\right.$$

$$Spatiotemporal\;model\;2\left\{ \begin{array}{l} logit\left(p_{it}\right)=\;\beta_{0}^{p}+\sum_{j}\beta_{j}^{p}\times Potential\;risk\;factors_{it,j}^{p}+\\ \kappa^{p}\times Rodent\;suitability_{it}^{p}+u_{i}^{p}+v_{i}^{p}+\gamma_{t\;}^{p}+\phi_{t}^{p}+\delta_{it}^{p}\\ \log\left(\mu_{it}\right)=\;\beta_{0}^{\mu }+\sum_{j}\beta_{j}^{\mu }\times Potential\;risk\;factors_{it,j}^{\mu }+\\ \kappa^{\mu }\times Rodent\;suitability_{it}^{\mu }+u_{i}^{\mu }+v_{i}^{\mu }+\gamma_{t}^{\mu }+\phi_{t}^{\mu }+\delta_{it}^{\mu } \end{array}\right.$$

To compare the fit of the models, the Watanabe-Akaike information criterion (WAIC) was used. WAIC offers the advantage of averaging over the posterior distribution instead of relying on a single-point estimate, making it particularly valuable for models with hierarchical or mixed structures where point estimates may not be meaningful, even in cases involving singular models [47]. In general, a model with smaller WAIC value was better supported by data [47]. In this study, final model selection considered both WAIC values and the scientific relevance of covariates. Specifically, we prioritized models that allowed evaluation of our primary hypothesis regarding habitat suitability as a risk factor for scrub typhus incidence. Posterior means and 95% credible intervals (CrI) were reported, and model fit was evaluated spatially and temporally by plotting the fitted and predicted values of ST incidence.

Subgroup analyses by sex and age groups (0‐39, 40‐59, 60‐79, and ≥80 years of age) were conducted using ST case counts as the outcome and the log-transformed population as an offset. Information on woman farmer population was excluded when performing subgroup analysis according to gender, to avoid the possibility of overcorrection. Modeling was performed using R software (version 4.2.1; R Foundation for Statistical Computing) [35] (Multimedia Appendix 2).

#### Sensitivity Analysis

Sensitivity analysis was performed using a different spatial weight matrix constructed through a k-nearest neighbor approach, with k set at 5, to assess the robustness of the results. In addition, another sensitivity analysis was conducted using the number of ST cases as the outcome and log-transformed population as an offset.

### Ethical Considerations

This study received an exemption from the Institutional Review Board (IRB) of Korea University (IRB exemption number: KUIRB-2021-0237-02) as the data used in this study were fully anonymized and contained no personally identifiable information. The requirement for obtaining informed consent was waived as the study was observational in design. As the analysis used secondary data, no compensation was provided.

## Results

### Descriptive Analysis

**Figure 1.**
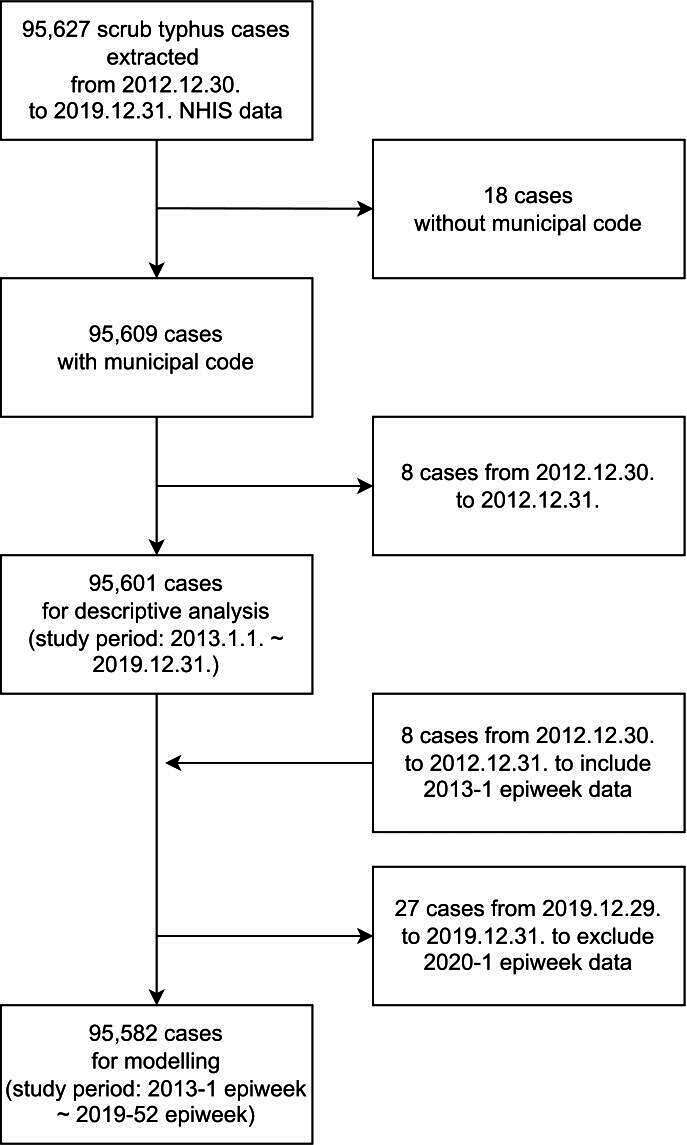
Flowchart of scrub typhus cases diagnosed with International Classification of Diseases, 10th Revision-Clinical Modification code A75.3, extracted from the National Health Insurance Service data in South Korea from 2013 to 2019. Epiweek: epidemiological week; NHIS: National Health Insurance Service in South Korea.

A total of 95,627 ST cases were extracted between December 30, 2012, and December 31, 2019 from the NHIS data at the municipal level. For the descriptive analysis, 95,601 patients with ST were considered, and for the modeling, 95,582 patients with cases that corresponded to the epiweek-based study period (from the first epiweek of 2013 to the 52^nd^ epiweek of 2019) were included (Figure 1).

Between 2013‐2019, ST cases (18,822 in 2013; 7770 in 2019) and incidence per 100,000 population (36.8 in 2013; 15.0 in 2019) decreased (Table S3 in Multimedia Appendix 1, [21]). Although a marginal increase was observed in 2016 (cases: 17,086; incidence: 33.1), the cases decreased by around 58.7% between 2013‐2019, with a 59.02% decrease in incidence over the same period. The ST incidence per 100,000 population was consistently higher in women than in men between 2013‐2019 (men: 22.0, women: 30.9). The ≥80 years of age group had the highest incidence (94.6), followed by the 60‐79 (79.4), 40‐59 (24.1), and 0‐39 age group (4.96; Table S4 in Multimedia Appendix 1) [21].

Baseline information for each variable is provided in Table 2. The average number of ST cases in 2013‐2019 at the municipal level and epiweek was 1.0 (SD 3.5) and the coefficient of variance (CV) was 3.4, suggesting regional and temporal variations. The CV of potential risk factors ranged from 0.2 to 1.1, indicating lower regional and temporal variation than ST incidence per 100,000 population (CV=4.2).

**Table 2.** Summary statistics of scrub typhus incidence and potential risk factors by municipality and epidemiological week in South Korea, 2013-2019.

Variables	Mean (SD)		Minimum	Median (Q1-Q3)	Maximum	Coefficient of variance
Scrub typhus						
Cases (n)	1.0 (3.5)		0.0	0.0 (0.0-1.0)	81.0	3.4
Incidence per 100,000 population	1.0 (4.1)		0.0	0.0 (0.0-0.3)	105.7	4.2
Potential risk factors						
Maximum rodent suitability	0.8 (0.2)		0.1	0.8 (0.7-0.9)	1.0	0.2
Financial independence (%)	28.6 (14.9)		7.3	24.2 (16.3-38.9)	75.9	0.5
Forest area (*m*^2^)	255,633,212.9 (287,531,335.5)		0.0	178,314,785.4 (18,240,087.4- 405,361,824.9)	1,532,013,213.1	1.1
Dry field farming area (*m*^2^)	30,624,701.2 (32,395,574.9)		0.0	23,566,365.5 (2,706,313.0- 52,257,727.8)	210,272,858.0	1.1
Woman farmer population (n)	5,153.8 (4,617.4)		147.0	4286.0 (1068.0-7939.0)	32,932.0	0.9
Offset						
Population (n)	206,370.1 (162,965.9)		9617.0	175,277.0 (59,061.0-314,110.0)	863,720.0	0.8

**Figure 2.**
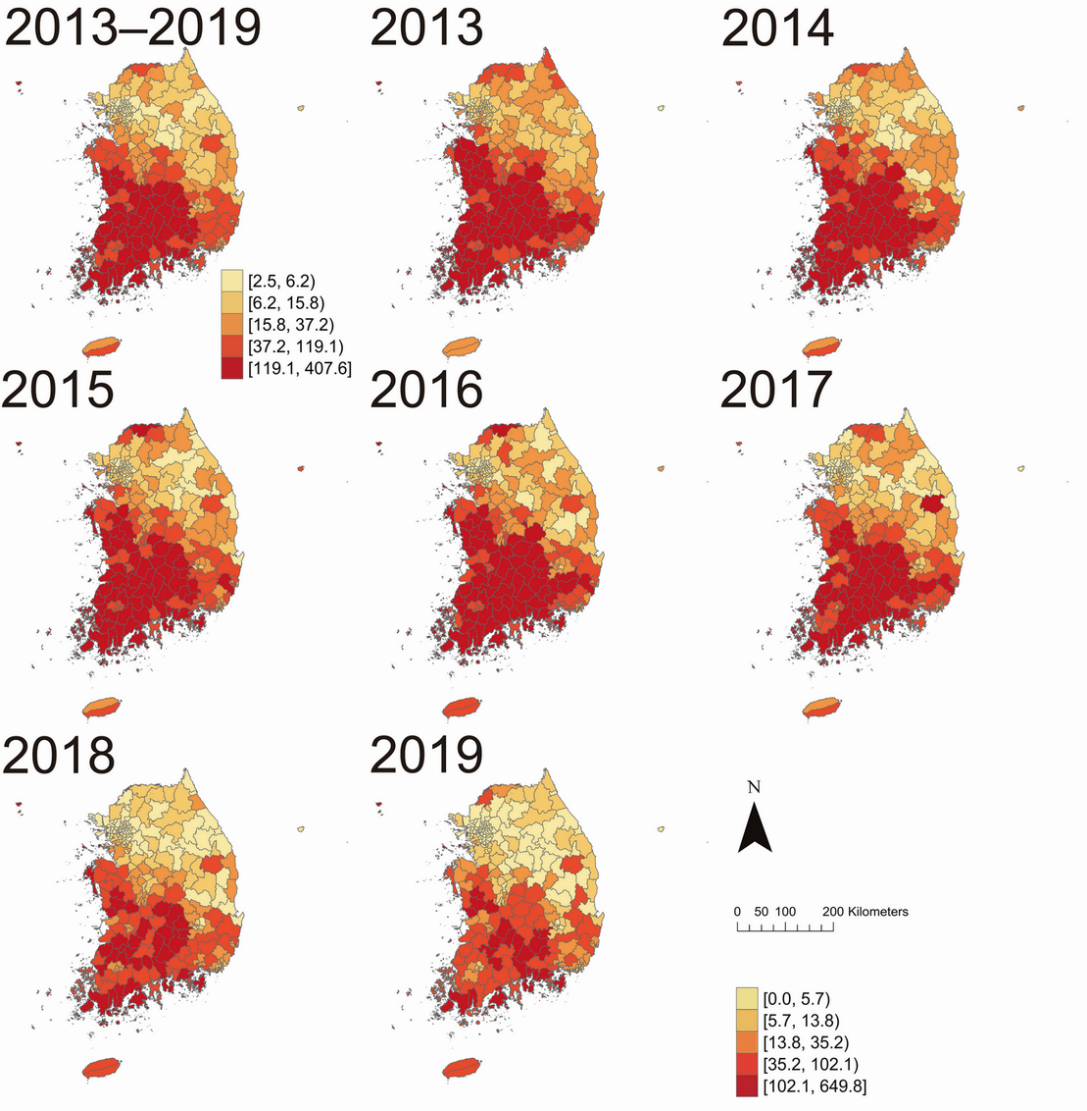
Annual incidence of scrub typhus per 100,000 population in 250 municipalities across South Korea from 2013 to 2019. Each panel represents a specific year, with the top-left panel showing the cumulative incidence from 2013 to 2019. Colors represent incidence rate ranges, where darker red indicates higher incidence and light yellow indicates lower incidence.

According to the regional distribution map of ST incidence per 100,000 population in 2013‐2019, the southwestern parts of South Korea had higher ST incidence (119.1‐407.6 incidence per 100,000 population) than northeastern parts (Figure 2). Regardless of the year, the ST incidence was the highest in the southwestern region of South Korea. This trend was consistent, regardless of gender (Figure S1 in Multimedia Appendix 1) or age group (Figure S2 in Multimedia Appendix 1). However, over time, the number of municipalities with the highest ST incidence quintile (102.1‐649.8 incidence per 100,000 population) decreased.

Among all the study participants, the results of the Global Moran’s *I* statistics indicated that ST incidence had a positive spatial autocorrelation (*I*=0.600; *P*=.01, Table S5 in Multimedia Appendix 1). Positive spatial autocorrelation was consistently reported in 2013‐2019, with 2015 having the strongest index value (*I*=0.594; *P*=.01). Positive spatial autocorrelation was consistently reported regardless of gender (men, *I*=0.600, *P*=.01; women, *I*=0.590, *P*=.01) and age group (age 0‐39 years, *I*=0.564, *P*=.01; age 40‐59 years, *I*=0.630, *P*=.01; age 60‐79 years, *I*=0.649, *P*=.01; age≥80 years, *I*=0.622, *P*=.01).

**Figure 3.**
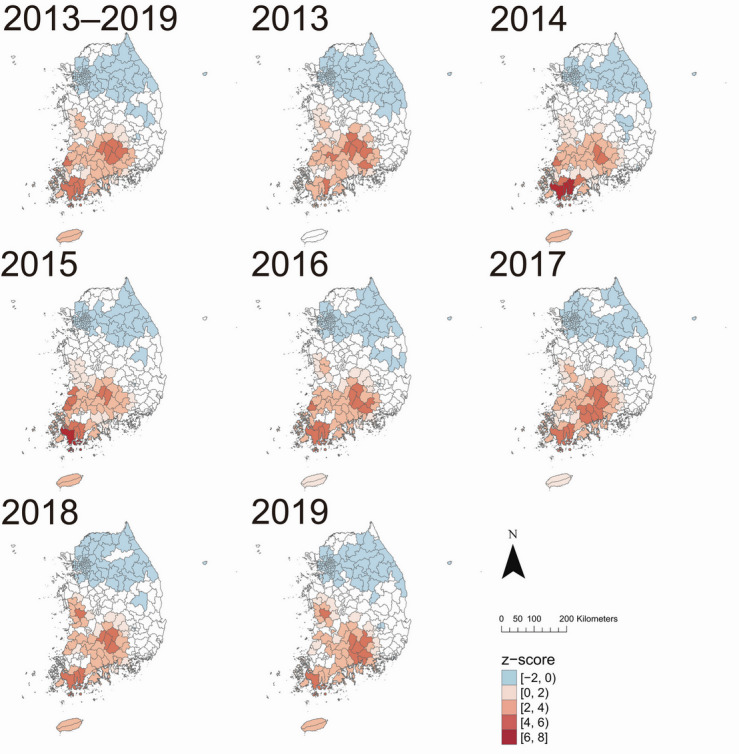
Spatial clusters of scrub typhus incidence per 100,000 population identified using Getis-Ord $G_{i}^{*}$ analysis across 250 municipalities in South Korea, annually from 2013 to 2019 and for the overall period. Red shades indicate hot spots (high positive *z* score), and blue shades indicate cold spots (low negative *z* score). Color intensity reflects the magnitude of the *z* score resulted from Getis-Ord $G_{i}^{*}$ analysis.

The Getis-Ord $G_{i}^{*}$ analysis on ST incidence per 100,000 population consistently detected hot and cold spots in the southwestern and northeastern parts of South Korea, respectively, between 2013‐2019 (Figure 3). The trend persisted, regardless of gender (Figure S1 in Multimedia Appendix 1) and age group (Figure S2 in Multimedia Appendix 1).

**Figure 4.**
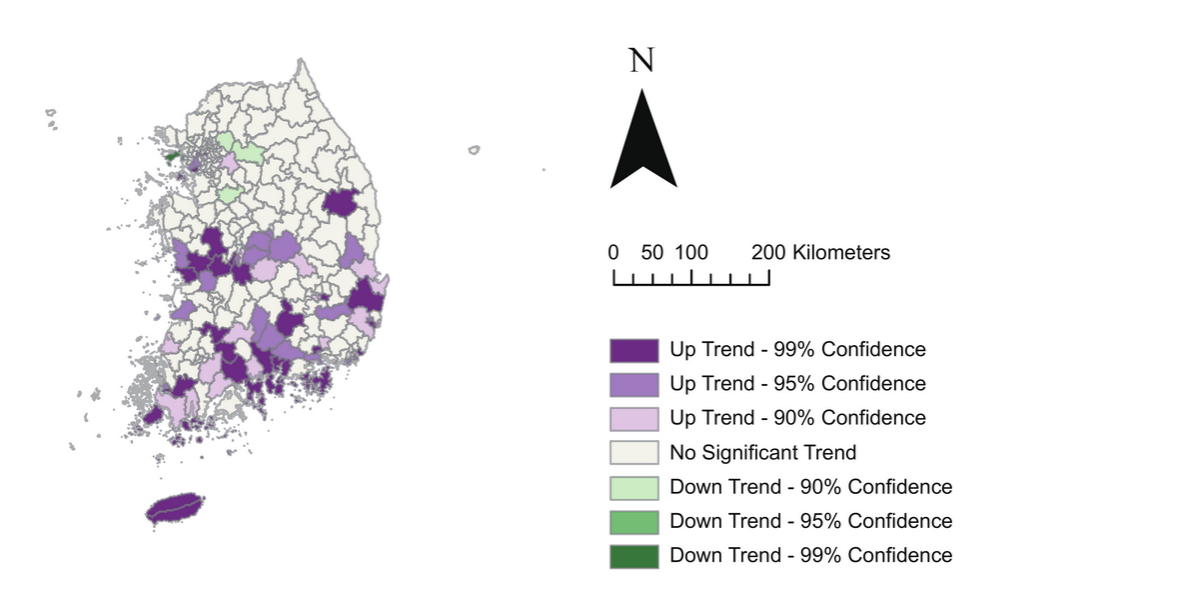
Hot and cold spot trends of scrub typhus incidence per 100,000 population across 250 municipalities in South Korea, from 2013 to 2019, visualized using a 2-dimensional representation of a space-time cube. Purple shades represent up trends (hot spot trends), and green shades indicate down trends (cold spot trends), each categorized by 90%, 95%, and 99% confidence levels. White areas indicate no significant trend.

The HCT results for the total population indicated increasing trends in municipalities across the southern and mid regions of South Korea Figure 4. Municipalities showing increasing trends were also observed in the northwestern part, specifically around the capital region. This pattern was consistent for both genders (Figure S1 in Multimedia Appendix 1), with the southern island of Jeju-do consistently exhibiting an upward trend, regardless of gender and age group. However, the trend varied according to the age group (Figure S2 in Multimedia Appendix 1). Specifically, in the 0‐39 years age group, municipalities with increasing trends were scattered throughout the country, but the upward trend was more clearly observed in the capital region compared with other age groups. For the 40‐59, 60‐79, and ≥80 years age groups, municipalities with increasing trends were more concentrated in the southern and mid regions.

### Modeling

The results of multivariable Bayesian models on ST incidence are presented in Table 3 and Table S6 in Multimedia Appendix 1. Overall, spatiotemporal models demonstrated better fit compared with spatial and temporal models, with lower WAIC values. Among the spatiotemporal models, model 0 had the lowest WAIC value (75,014.8), followed by model 1 (75,019.1), and model 2 (75,037.2). However, given its ability to incorporate relevant covariates and test the primary hypothesis, spatiotemporal model 2 was retained as the model of interest, despite having a slightly higher WAIC than model 0. Sensitivity analysis results also showed that the spatiotemporal model had lower WAIC than spatial and temporal models (Table S7 in Multimedia Appendix 1). By comparing the maps (Figure S3 in Multimedia Appendix 1) and time-series plot (Figure S4 in Multimedia Appendix 1) between the observed and predicted ST incidences using spatiotemporal model 2, the distributions had similarities.

**Table 3.** Results of multivariable Bayesian models for age-standardized scrub typhus incidence in South Korea, 2013 to 2019, presented as β coefficients (95% credible intervals).

Model fit metric and variables	Spatial model 2^a^	Temporal model 2^b^	Spatiotemporal model 2^c^
WAIC^d^	211,792.6	115,177.7	75,037.2
Maximum rodent suitability, β coefficient (95% CrI^e^)	0.320 (0.075-0.564)	0.493 (0.477-0.509)	0.618 (0.425-0.812)
Financial independence (%), β coefficient (95% CrI)	−0.486 (−0.553 to −0.419)	−0.722 (−0.736 to −0.709)	−0.304 (−0.445 to −0.163)
Forest area (*m*^2^), β coefficient (95% CrI)	0.604 (0.289-0.930)	0.086 (0.074-0.099)	0.171 (−0.076 to 0.421)
Dry field farming area (*m*^2^), β coefficient (95% CrI)	−0.719 (−1.009 to −0.423)	−0.184 (−0.199 to −0.169)	0.040 (−0.228 to 0.311)
Woman farmer population (n), β coefficient (95% CrI)	1.582 (1.525-1.640)	0.272 (0.258-0.287)	0.081 (−0.104 to −0.266)

a Model including spatial terms and potential risk factors, including maximum rodent suitability.

b Model including temporal terms and potential risk factors, including maximum rodent suitability.

c Model including spatial and temporal terms, interaction term of spatial and temporal structured terms, and potential risk factors, including maximum rodent suitability.

d WAIC: Watanabe-Akaike information criterion.

e CrI: credible interval.

According to the results of spatiotemporal model 2, maximum rodent suitability (β coefficient=0.618, 95% credible interval [CrI] 0.425‐0.812) was positively associated with ST incidence, while financial independence was negatively associated with ST incidence (β coefficient=−0.304, 95% CrI −0.445 to −0.163).

The subgroup analysis results by gender followed the trends of main analyses, except for forest area having a positive association (Table S8 in Multimedia Appendix 1). The results of the Bayesian spatiotemporal model differed by age group. Table 4 presented the results of the spatiotemporal models by age group. Regardless of age group, financial independence had negative and forest area had positive association with ST incidence. However, the incidence of the 40‐59, 60‐79, and ≥80 years of age group had positive association with maximum rodent suitability, while the incidence of 0‐39 years of age did not have a significant association with rodent suitability (β coefficient=0.028, 95% CrI −0.072 to 0.126). Only the incidence of the 0‐39 years of age group had a negative association with the women farmer population (β coefficient=−0.115, 95% CrI −0.223 to −0.006). The sensitivity analysis results followed the trends of main analyses, while forest area had significant association (Tables S7 and S9 in Multimedia Appendix 1) and women farmer population did not have significant association with ST incidence in the 0‐39 year age group like main analysis results (Table S9 in Multimedia Appendix 1).

**Table 4.** Results of multivariable Bayesian spatiotemporal models for scrub typhus incidence stratified by age group in South Korea, 2013 to 2019.

Model fit metric and variables	Age group (years)			
0‐39	40-59	60‐79	≥80
Participants, n (%)	8438 (8.8)	28,930 (30.3)	48,129 (50.3)	10,104 (10.6)
WAIC^a^	15,647.3	39,939.7	54,357.4	17,180.7
Maximum rodent suitability, β coefficient (95% CrI^b^)	0.028 (−0.072‐0.126)	0.149 (0.052‐0.246)	0.223 (0.127‐0.319)	0.099 (0.017‐0.180)
Financial independence (%), β coefficient (95% CrI)	−0.350 (−0.431 to −0.268)	−0.262 (−0.329 to −0.194)	−0.206 (−0.269 to −0.143)	−0.136 (−0.199 to −0.072)
Forest area (*m*^2^), β coefficient (95% CrI)	0.472 (0.332‐0.613)	0.308 (0.180‐0.436)	0.177 (0.052‐0.302)	0.174 (0.080‐0.268)
Dry field farming area (*m*^2^), β coefficient (95% CrI)	0.011 (−0.139 to 0.161)	−0.037 (−0.174 to 0.101)	−0.034 (−0.168 to 0.103)	−0.012 (−0.115 to 0.091)
Woman farmer population (n), β coefficient (95% CrI)	−0.115 (−0.223 to −0.006)	0.036 (−0.046 to 0.118)	0.066 (−0.011 to 0.143)	−0.077 (−0.164 to 0.009)

a WAIC: Watanabe-Akaike information criterion.

b CrI: credible interval.

## Discussion

### Principal Findings

This study examined the demographic, spatial, and temporal distributions of the incidence of ST. Notably, ST incidence consistently exhibited spatial hotspots in the southwestern region of South Korea, regardless of year, sex, or age. This pattern aligns with previous findings and can be attributed to the ecological characteristics of the southwestern region, which is characterized by fertile lands conducive for rodent and mite habitats [48].

Moreover, HCT analysis revealed a consistent increasing trend in the southern and mid regions, despite a declining trend in incidence. A similar spatial expansion of ST cases from the southwestern to northeastern regions was noted between 2001‐2006 [48], which was likely attributed to the effects of climate change, particularly rising temperatures that enhanced vector survival, expanded their geographic range, and accelerated pathogen growth [49]. However, variations in ST trends were observed when age groups were considered. The younger age group (0‐39 years) exhibited scattered municipalities with increasing trends across the country, whereas older age groups (40‐59, 60‐79, and ≥80 years) displayed more concentrated increases in the southern and mid regions. This suggests that age-specific factors play a role in ST transmission dynamics and necessitate tailored prevention strategies based on specific demographic segments.

The current analysis further revealed the importance of incorporating both spatial and temporal dimensions in understanding the epidemiology of ST. Based on the WAIC, models with spatiotemporal interaction term (Spatiotemporal model 0, WAIC=75,014.8; Spatiotemporal model 1, WAIC=75,019.1; Spatiotemporal model 2, WAIC=75,037.2, Table 3, Table S7 in Multimedia Appendix 1) demonstrated a better fit than those with only spatial or temporal terms, regardless of including potential risk factors. This pattern was consistent in a sensitivity analysis using the number of ST cases as the outcome and log-transformed population as an offset (Table S7 in Multimedia Appendix 1), further confirming the benefit of modeling spatiotemporal interaction. This underscores the need for a comprehensive approach that considers both the spatial and temporal dimensions when investigating ST patterns. In particular, the ST data showed distinct temporal patterns in each area that were inconsistent with those in other regions, and the spatial patterns exhibited considerable variability (Figure S5 in Multimedia Appendix 1). This supports the necessity of including a space-time perspective in the analysis. However, as shown in another sensitivity analysis using a more complex spatial structure, convergence issues occurred in the INLA-based model (Table S9 in Multimedia Appendix 1). This highlights the choice of spatial weight matrix that can affect model convergence in complex Bayesian settings.

We revealed a significant association between ST incidence and specific factors. Our findings quantitatively investigated that municipalities with high rodent suitability were more likely to experience higher ST incidence, supporting previous studies [50-53]. High rodent density, particularly in areas with abundant hosts, contributes significantly to the transmission of *Orientia tsutsugamushi* [52]. In addition, climate variability and rodent abundance jointly influence scrub typhus transmission [53]. The nonsignificant but positive point coefficient observed in the 0‐39 years age group might be due to the smaller number of ST cases and reduced exposure to rodents at home or work. However, as rodent suitability is determined by environmental potential for rodent habitation than actual numbers, future studies should focus on monitoring rodent distribution across the nation and disseminating this information to the public. Additionally, research exploring the relationship between ST cases and the distribution of natural hosts based on this data is needed. Managing human-rodent interaction, as proposed by Sharma et al [54], could be a key strategy for reducing scrub typhus transmission, particularly through rodent habitat control and improved hygiene practices.

Furthermore, this study highlighted the impact of municipality financial status on ST incidence. Municipalities with lower financial independence were more likely to have higher ST incidence, which may reflect the characteristics of rural areas where agricultural and forest environments are common [55,56]. Although these regions may receive targeted support from central government programs, their ecological and occupational conditions likely contribute to the elevated risk.

Following the results of previous studies [8,57], our findings indicated that municipalities with more forest coverage had higher ST incidence in age- and gender–stratified models. Occupational exposure to forest environments, particularly among forestry workers, significantly elevates risk [57]. In addition, Zangpo et al [8] identified activities like cardamom harvesting and living near forests as key risk factors, highlighting the importance of forest proximity in ST transmission. Therefore, implementing targeted preventive strategies, including regular monitoring and enhanced protective education for forestry workers and residents near forested areas, is essential to mitigate future ST cases.

The association between the population of women farmers and ST incidence was mainly observed in the 0‐39 years age group, where women farmer population appeared to have a protective effect on ST incidence at the municipal level. This reflects contrasting trends of increasing ST incidence among men and the decline in the population of women farmers within this age group. From 2003 to 2019, ST incidence became more male-dominant in the 0‐39 years age group, reducing the gender disparity in ST incidence [21]. This age group exhibited a marked increase in ST incidence in the surrounding capital area (Figure S2 in Multimedia Appendix 1), where farming is less prevalent and the proportion of women farmers sharply declined (Figure S6 in Multimedia Appendix 1). Since women farmers have been the main target of ST prevention efforts due to their risk from agricultural work habits during harvest seasons [1,58,59], these findings may indicate a gap in preventive outreach to other at-risk populations, such as younger individuals engaging in nonfarming outdoor activities [60,61]. Considering the changing demographics in both farming populations and ST cases, updated mitigation strategies, such as redefining target populations in nontraditional rural areas, are necessary.

Although previous studies have suggested that dry field farming is associated with outdoor exposure and ST risk [10,33], no statistically significant association was observed in our models. This reflects regional variation in land use, ecological conditions, or population-level exposure, which warrants further investigation.

### Strengths and Limitations

To the best of our knowledge, this study is the first to investigate the spatiotemporal dynamics and risk factors of ST in South Korea in a One Health perspective. The considerable diversity observed in the municipal-level epidemic dynamics of ST underscores the necessity for a regional comprehension of the ST infection pattern. This understanding is crucial for the effective implementation of prevention and control strategies tailored to the burden of ST and its determinants at the municipal level. Furthermore, incorporating specific determinants of ST infection into these localized strategies or developing targeted group interventions is warranted. However, this study has some limitations. This study was an ecological study conducted at the municipal level, so the results should not be applied at the individual level. While ecological fallacy and unmeasured confounders may exist, a key advantage of our study is that the results can be directly translated into municipal-level ST interventions. Although our multivariable model enabled simultaneous assessment of multiple risk factors, it may not fully account for variable-specific causal pathways, including distinct confounders and effect modification. Nevertheless, the approach remains valuable insights into overall patterns and supports future hypothesis-driven research. Moreover, the NHIS data provided only municipal residential codes and lacked information on the bite site. As a result, spatial patterns, particularly among younger or more mobile populations, should be interpreted with caution. Further studies that incorporate bite-site information and target the entire nation are required. Although leisure activities such as hiking or camping may contribute to ST risk in younger populations [60], relevant municipal-level indicators were not available. Moreover, ST incidence remains the highest in older adults (Figure 4), despite increasing trends in recreational activity, which suggests that limited explanatory power of leisure exposure. Some popular recreational areas, including mountainous regions and forested areas like Gangwon-do, also showed low incidence (Figures2  3), possibly due to ecological factors such as climate and latitude affecting vector habits [3,8]. In addition, direct information on mite distribution across the country is unavailable. While vector surveillance programs such as Vector-net could serve as alternatives, their data were not publicly accessible. Therefore, partial compensation has been attempted by using information on rodent suitability, considering that rodents serve as natural hosts of mites and rodent density has been shown to be related to chigger mite distribution [62]. The construction of maximum rodent suitability also incorporated environmental factors, aligning with a One Health approach. Despite these limitations, these findings are expected to provide a foundation for addressing ST through evidence-based strategies based on examined hot spots and risk factors, grounded in a holistic approach that encompasses both One Health and spatiotemporal aspects.

### Conclusions

This study provides a detailed analysis of the spatiotemporal dynamics and risk factors of ST in South Korea from 2013 to 2019, emphasizing the importance of incorporating spatiotemporal aspects. The findings indicated positive spatial autocorrelation, with ST expanding from the southwest to the northeast regions. Municipalities with higher rodent suitability, greater forest coverage, and lower financial independence were more likely to experience increased ST incidence, with risk factors varying by age group. This study underscores the value of a holistic, spatiotemporal approach, highlighting the roles of rodent suitability, forest area, and socioeconomic factors in shaping ST dynamics. These findings may inform targeted public health intervention in Korea and provide broader insights applicable to other regions where similar ecological conditions and socioeconomic contexts exist. These insights provide a foundation for developing targeted public health interventions grounded in a One Health perspective to more effectively manage ST in South Korea and similar global settings.

## Supplementary material

10.2196/68437 Multimedia Appendix 1 Additional descriptive and spatiotemporal analyses of scrub typhus incidence, clusters, and risk factors in South Korea, 2013–2019.

10.2196/68437 Multimedia Appendix 2 R code for the Bayesian hurdle Poisson spatiotemporal analysis.

10.2196/68437 Checklist 1 STROBE (Strengthening the Reporting of Observational Studies in Epidemiology) checklist.

## Abbreviations

CrI: credible interval
CV: coefficient of variance
DEM: digital elevation model
EVI: enhanced vegetation index
HCT: hot and cold spot trend
iCAR: intrinsic conditional auto-regressive
*ICD-10-*CM code: *International Classification of Diseases, Tenth Revision*, Clinical Modification code
INLA: Integrated Nested Laplace Approximation
LST: land surface temperature
MaxEnt: maximum entropy model
MODIS: moderate resolution imaging spectroradiometer
NHIS: National Health Insurance Service
ST: scrub typhus
WAIC: Watanabe-Akaike information criterion
