# Supplementary material for: Distribution and Risk Factors of Scrub Typhus in South Korea, From 2013 to 2019: Bayesian Spatiotemporal Analysis
Source: JMIR Public Health Surveill. 2025 Sep 10;11:e68437. doi: 10.2196/68437 (PMC12422590; doi:10.2196/68437)
Supplement: Multimedia Appendix 1 [file publichealth-v11-e68437-s001.docx]

## Figures


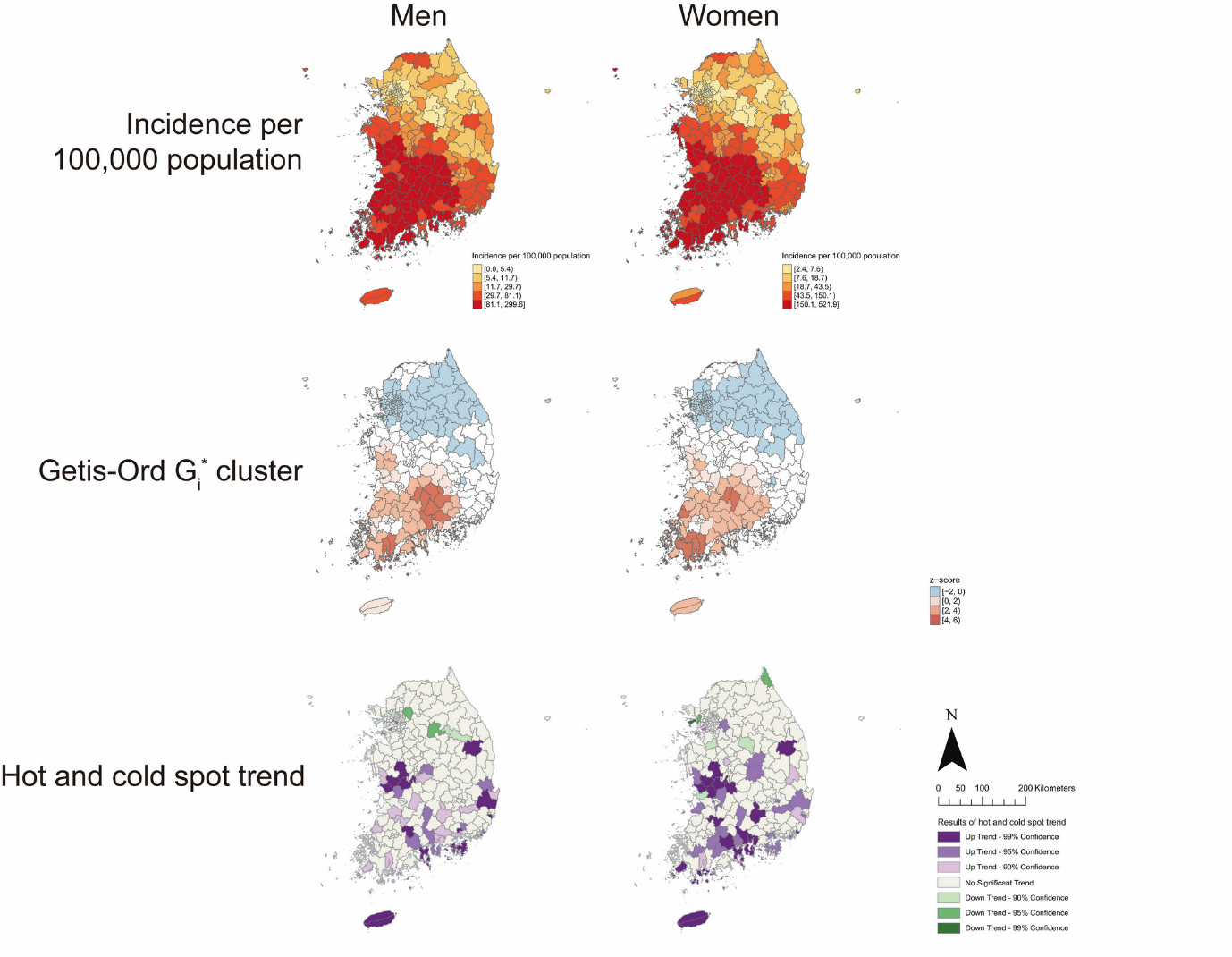


Supplementary Figure S1. Scrub typhus incidence per 100,000 population, clusters identified by Getis-Ord $G_{i}^{*}$ analysis, and results of hot and cold spot trend by visualizing a space-time cube in two dimensions across 250 municipalities in South Korea from 2013 to 2019 by gender. For choropleth maps with incidence, colors represent incidence rate ranges, where darker red indicates higher incidence and light yellow indicates lower incidence. For the results of Getis-Ord $G_{i}^{*}$ analysis, red shades indicate hot spots (high positive z -score), and blue shades indicate cold spots (low negative z-score). Color intensity reflects the magnitude of the z-score resulted from Getis-Ord $G_{i}^{*}$ analysis. For the results of hot and cold spot trend analysis, purple shades represent up trends (hot spot trends), and green shades indicate down trends (cold spot trends), each categorized by 90, 95, and 99% confidence levels. White areas indicate no significant trend.


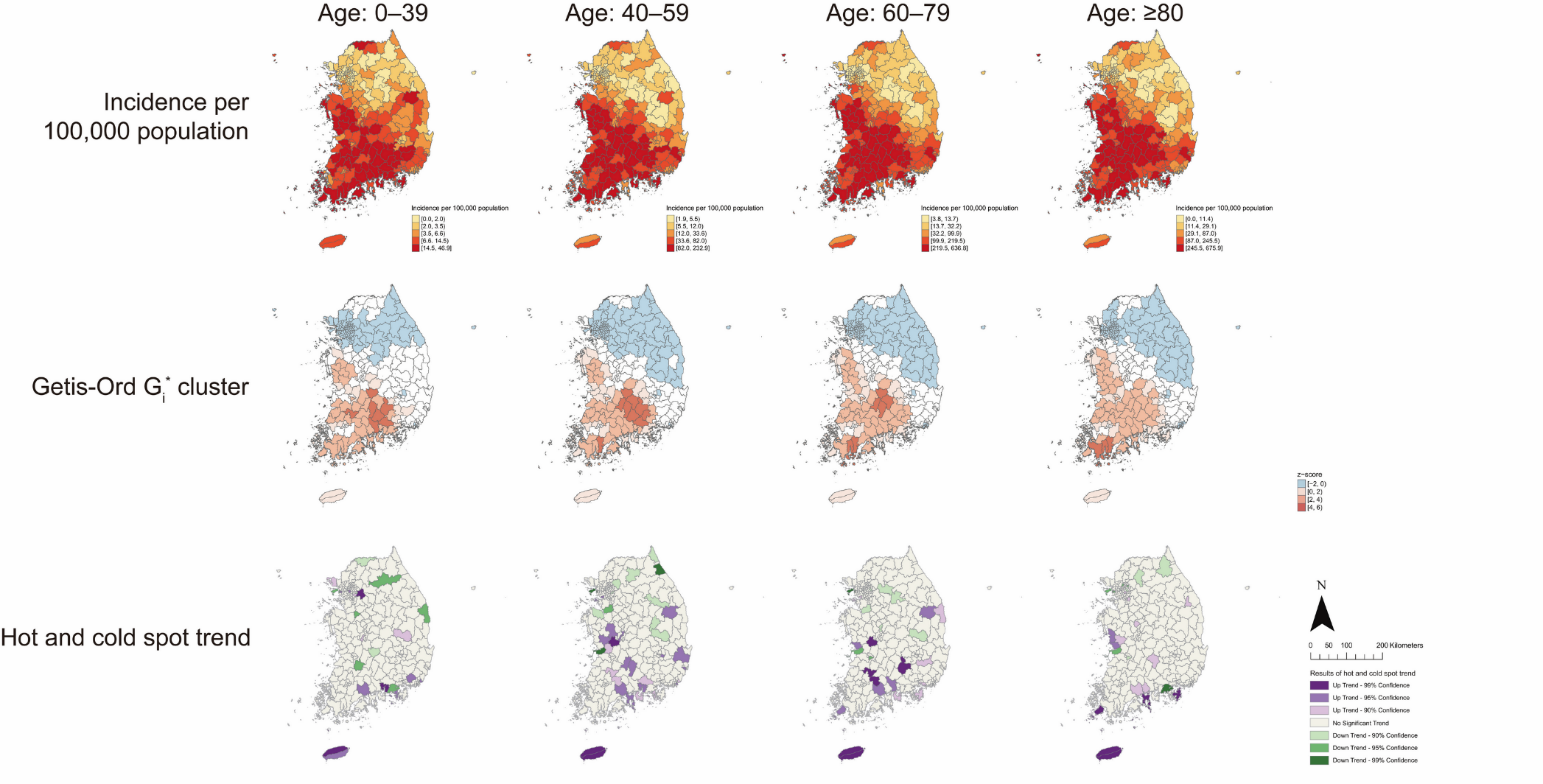


Supplementary Figure S2. Scrub typhus incidence per 100,000 population, clusters identified by Getis-Ord $G_{i}^{*}$ analysis, and results of hot and cold spot trend by visualizing a space-time cube in two dimensions across 250 municipalities in South Korea from 2013 to 2019 by age group (0–39, 40–59, 60–79, and ≥80 years). For choropleth maps with incidence, colors represent incidence rate ranges, where darker red indicates higher incidence and light yellow indicates lower incidence. For the results of Getis-Ord $G_{i}^{*}$ analysis, red shades indicate hot spots (high positive z -score), and blue shades indicate cold spots (low negative z-score). Color intensity reflects the magnitude of the z-score resulted from Getis-Ord $G_{i}^{*}$ analysis. For the results of hot and cold spot trend analysis, purple shades represent up trends (hot spot trends), and green shades indicate down trends (cold spot trends), each categorized by 90, 95, and 99% confidence levels. White areas indicate no significant trend.


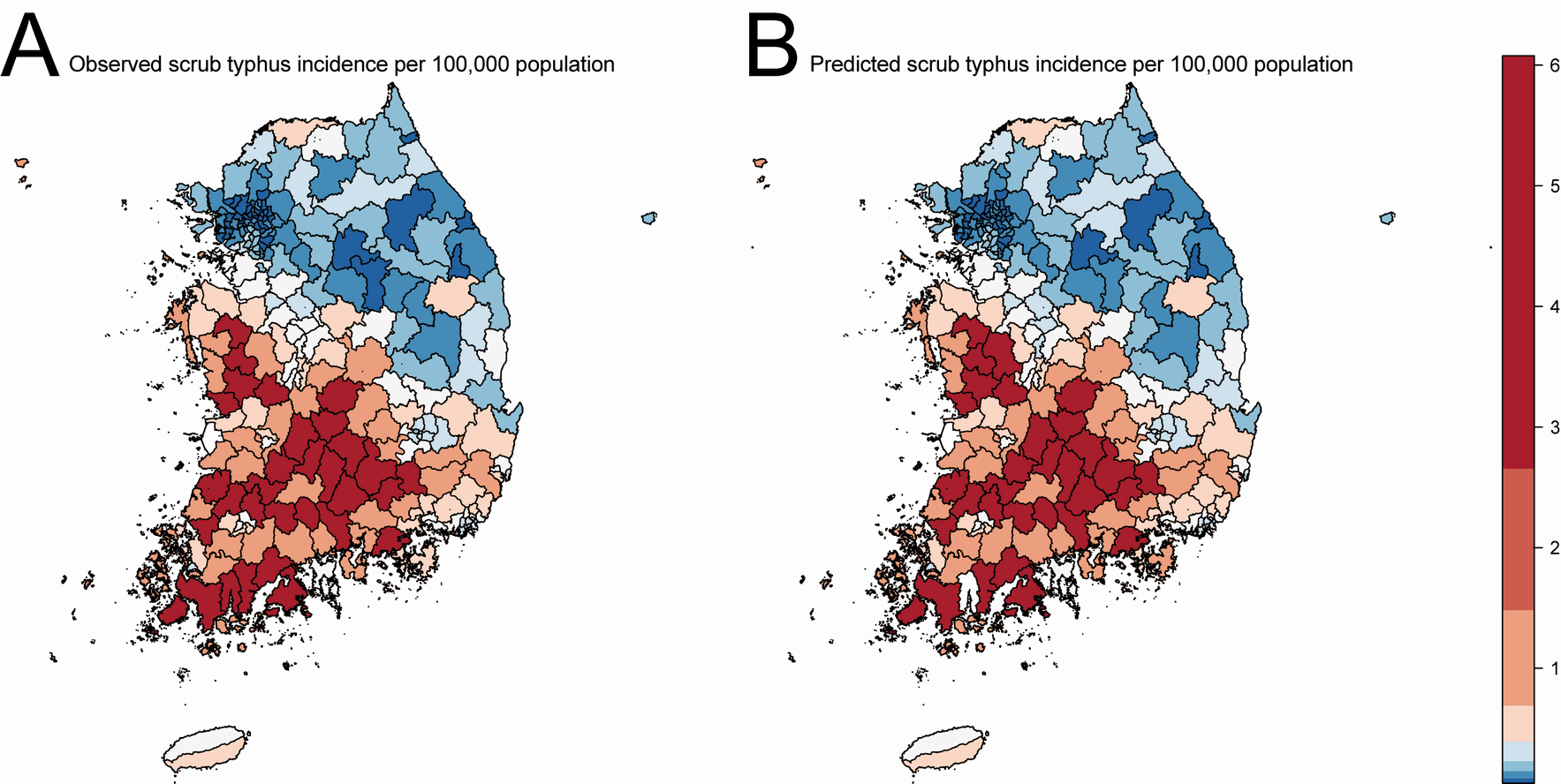


Supplementary Figure S3. Maps of observed scrub typhus incidence and of predicted incidence across 250 municipalities in South Korea by the Bayesian hurdle Poisson spatiotemporal model 2, which included spatial and temporal terms, interaction term of spatial and temporal structured terms, and potential risk factors, including maximum rodent suitability. Colors represent incidence rate ranges, where darker red indicates higher observed (or predicted) incidence and darker blue indicates lower observed (or predicted) Panel A shows observed values and Panel B shows model-predicted values.
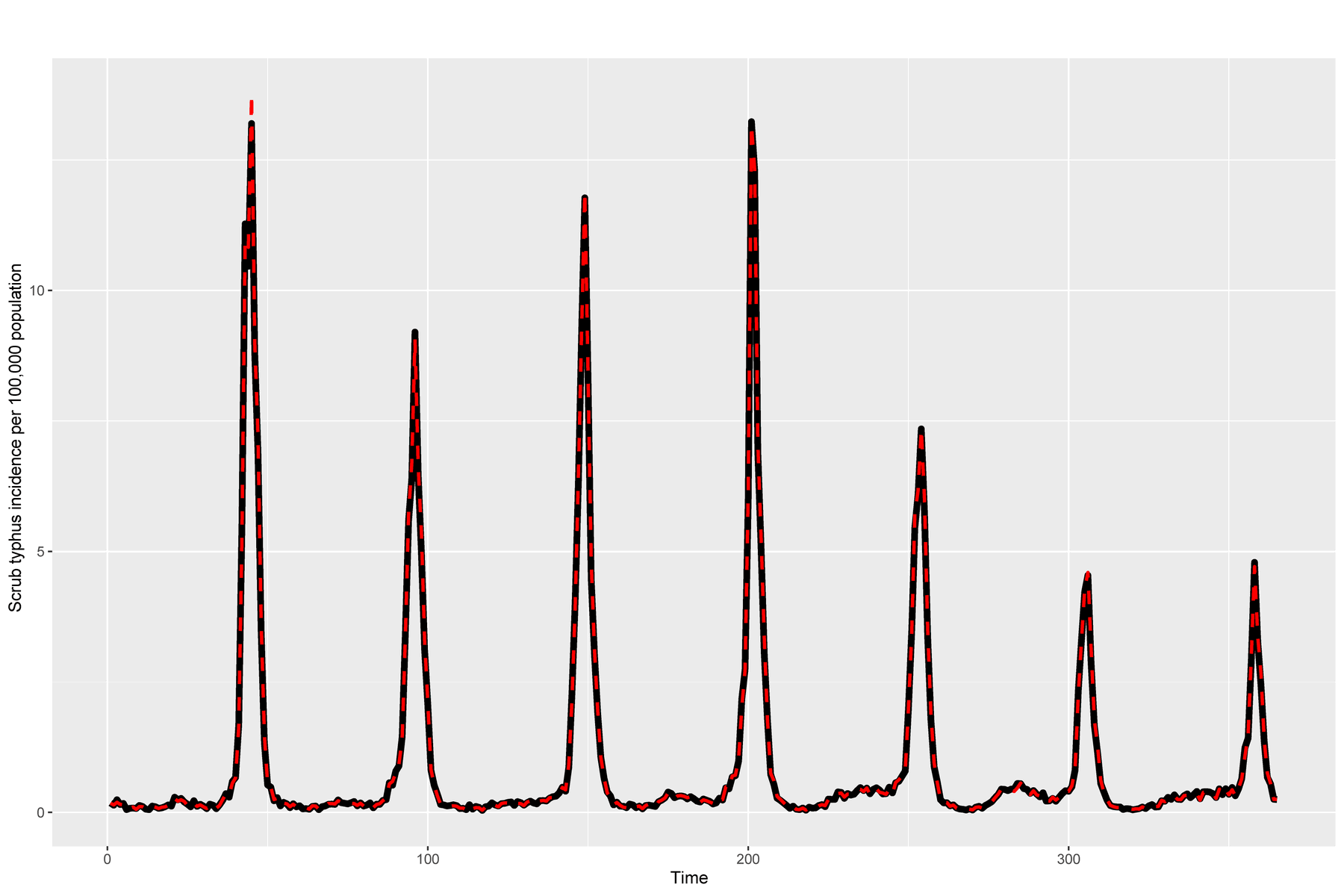


Supplementary Figure S4. Time-series plot of observed and predicted scrub typhus incidence per 100,000 population by the Bayesian hurdle Poisson spatiotemporal model 2, which included spatial and temporal terms, interaction term of spatial and temporal structured terms, and potential risk factors, including maximum rodent suitability. The Black line represents the observed values, and the red dashed line represents predicted values*.*


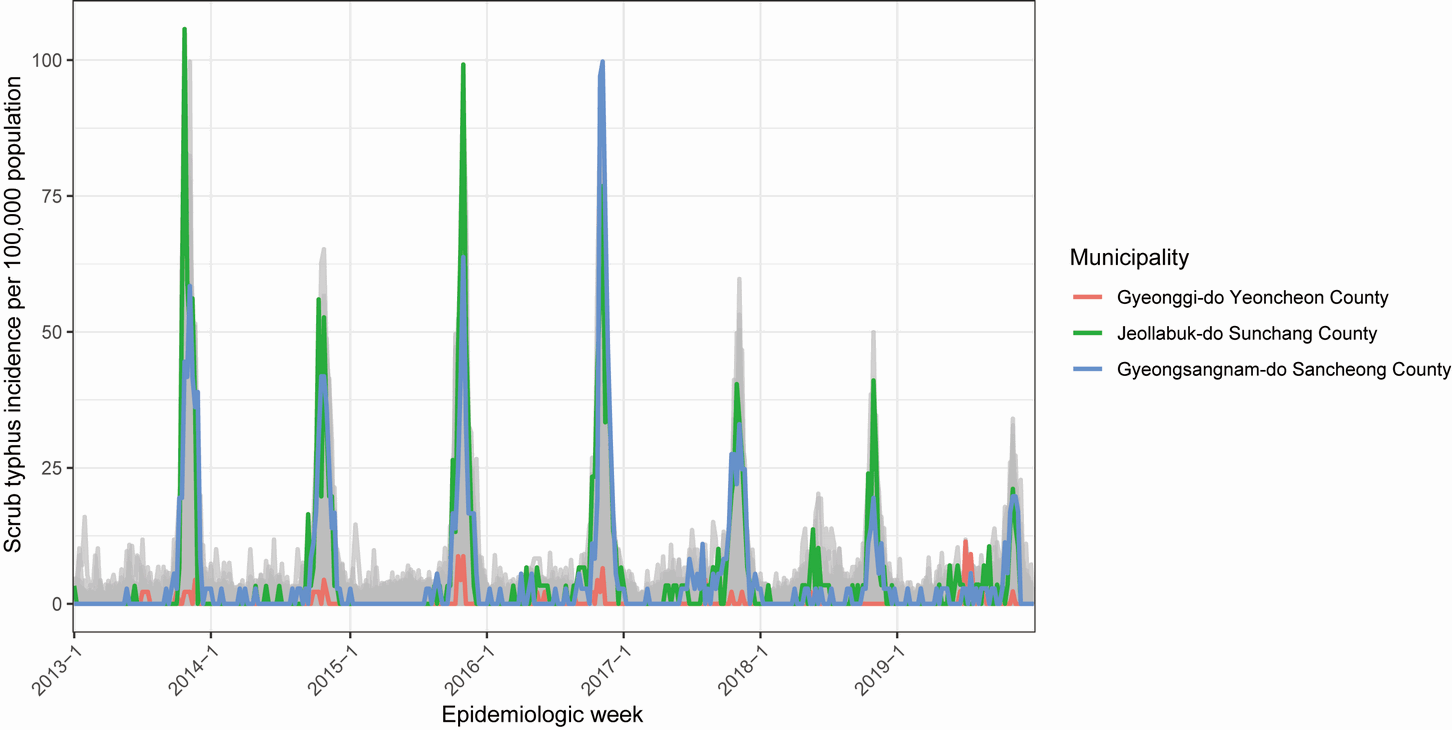


Supplementary Figure S5. Weekly observed scrub typhus incidence per 100,000 population from the first epidemiologic week in 2013 to 52^nd^ epidemiologic week in 2019 by 250 municipalities. Each line refers to each municipality. The red, green, and blue lines highlight Yeoncheon (Gyeonggi-do), Sunchang (Jeollabuk-do), and Sancheong (Gyeongsangnam-do), respectively. Other scrub typhus incidences of 247 municipalities are represented in grey lines.


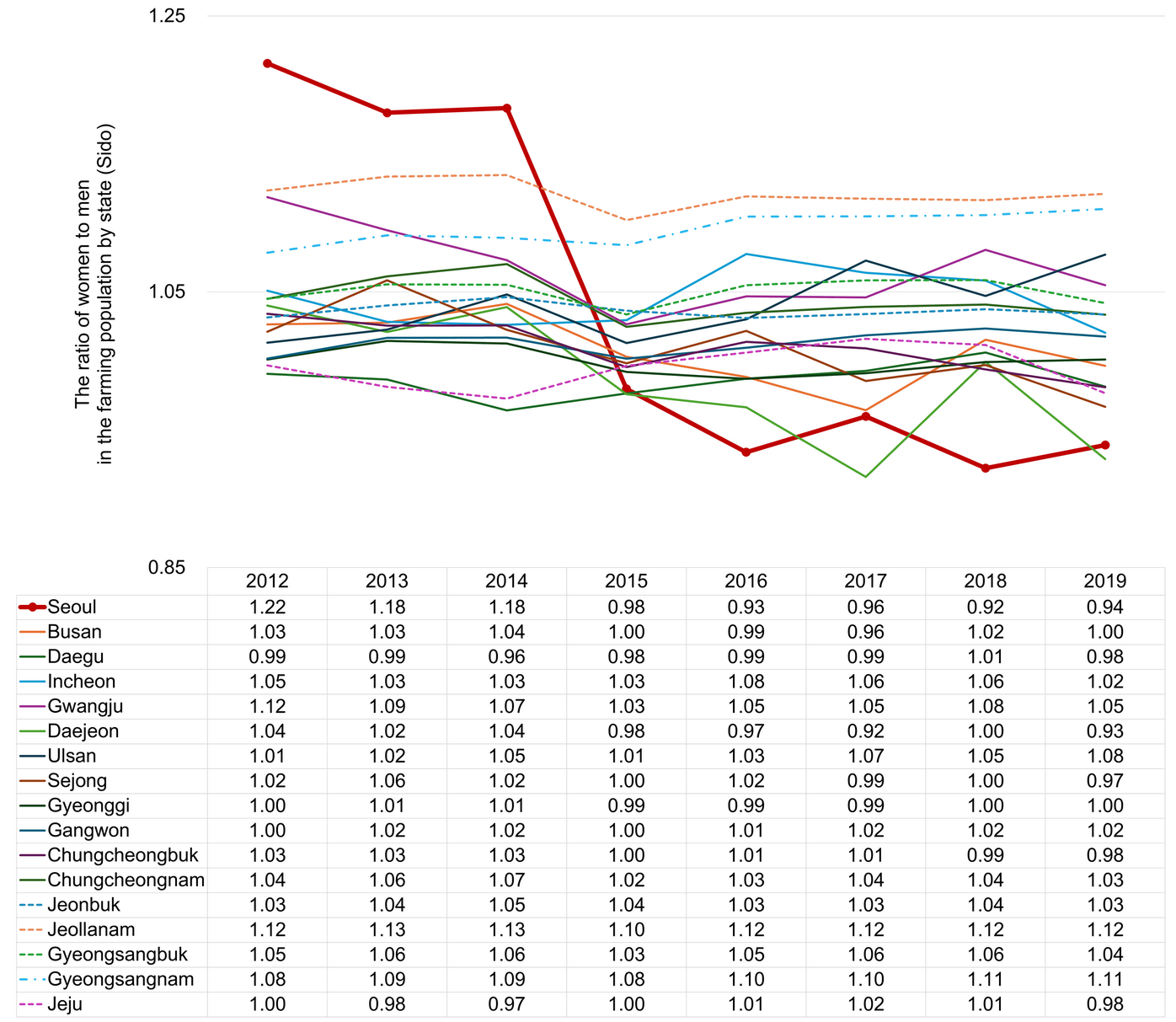


Supplementary Figure S6. Temporal trends in the ratio of women to men in the farming population by year and state (Sido) from 2012 to 2019. Each colored line represents one state (Sido).

## Tables

Supplementary Table S1. Results of univariable linear regression analyses assessing associations between potential risk factors and age-standardized scrub typhus incidence in South Korea, 2013 to 2019.

|  | Coefficient | P-value |
| --- | --- | --- |
| Maximum rodent suitability | 0.327 | <0.001 |
| Financial independence (%) | -0.366 | <0.001 |
| Forest area ($m^{2}$) | 0.184 | <0.001 |
| Dry field farming area ($m^{2}$) | 0.218 | <0.001 |
| Woman farmer population (N) | 0.353 | <0.001 |

Supplementary Table S2. Results of multivariable linear regression analyses assessing associations between potential risk factors and age-standardized scrub typhus incidence in South Korea, 2013 to 2019.

|  | Coefficient | P-value | Variance inflation factors |
| --- | --- | --- | --- |
| Maximum rodent suitability | 0.159 | <0.001 | 1.30 |
| Financial independence (%) | -0.344 | <0.001 | 1.25 |
| Forest area ($m^{2}$) | -0.032 | 0.026 | 2.22 |
| Dry field farming area ($m^{2}$) | -0.269 | <0.001 | 4.23 |
| Woman farmer population (N) | 0.431 | <0.001 | 3.29 |

Supplementary Table S3. Annual scrub typhus case counts and incidence per 100,000 population in South Korea, 2013 to 2019.

|  | Case count, N (%) | | | Incidence per 100,000 population | | |
| --- | --- | --- | --- | --- | --- | --- |
| Year | Total | Men | Women | Total | Men | Women |
| 2013 | 18,822 (100.0) | 7,742 (41.1) | 11,080 (58.9) | 36.8 | 30.3 | 43.4 |
| 2014 | 14,063 (100.0) | 5,705 (40.6) | 8,358 (59.4) | 27.4 | 22.2 | 32.6 |
| 2015 | 15,719 (100.0) | 6,286 (40.0) | 9,433 (60.0) | 30.5 | 24.4 | 36.6 |
| 2016 | 17,086 (100.0) | 7,191 (42.1) | 9,895 (57.9) | 33.1 | 27.8 | 38.3 |
| 2017 | 13,262 (100.0) | 5,608 (42.3) | 7,654 (57.7) | 25.6 | 21.7 | 29.5 |
| 2018 | 8,879 (100.0) | 3,769 (42.4) | 5,110 (57.6) | 17.1 | 14.6 | 19.7 |
| 2019 | 7,770 (100.0) | 3,387 (43.6) | 4,383 (56.4) | 15.0 | 13.1 | 16.9 |
| Total | 95,601 (100.0) | 39,688 (41.5) | 55,913 (58.5) | 26.5 | 22.0 | 30.9 |

Supplementary Table S4. Annual scrub typhus case counts and incidence per 100,000 population stratified by age group in South Korea, 2013 to 2019.

|  | Case count, N (%) | | | | | Incidence per 100,000 population | | | |
| --- | --- | --- | --- | --- | --- | --- | --- | --- | --- |
| Year | 0–39 years of age | 40–59 years of age | 60–79 years of age | ≥80 years of age | | 0–39 years of age | 40–59 years of age | 60–79 years of age | ≥80 years of age |
| 2013 | 1,653 (8.8) | 6,435 (34.2) | 9,205 (48.9) | 1,529 (8.1) | | 6.49 | 38.0 | 122.0 | 127.9 |
| 2014 | 1,275 (9.1) | 4,423 (31.5) | 7,020 (49.9) | 1,345 (9.6) | | 5.09 | 25.8 | 89.4 | 103.9 |
| 2015 | 1,335 (8.5) | 4,671 (29.7) | 8,038 (51.1) | 1,675 (10.7) | | 5.41 | 27.2 | 97.4 | 119.0 |
| 2016 | 1,511 (8.8) | 5,176 (30.3) | 8,637 (50.6) | 1,762 (10.3) | | 6.21 | 30.1 | 100.2 | 116.0 |
| 2017 | 1,217 (9.2) | 3,864 (29.1) | 6,692 (50.5) | 1,489 (11.2) | | 5.09 | 22.5 | 74.1 | 91.4 |
| 2018 | 783 (8.8) | 2,389 (26.9) | 4,535 (51.1) | 1,172 (13.2) | 3.33 | | 14.0 | 48.0 | 67.1 |
| 2019 | 664 (8.5) | 1,972 (25.4) | 4,002 (51.5) | 1,132 (14.6) | | 2.89 | 11.6 | 40.4 | 60.0 |
| Total | 8,438 (8.8) | 28,930 (30.3) | 48,129 (50.3) | 10,104 (10.6) | | 4.96 | 24.1 | 79..4 | 94.6 |

Supplementary Table S5. Results of Global Moran’s *I* statistics assessing spatial autocorrelation of scrub typhus incidence per 100,000 population in South Korea, 2013 to 2019, stratified by gender, age group, and year.

|  | Total population | Men | Women | 0–39 years of age | 40–59 years of age | 60–79 years of age | ≥80 years of age |
| --- | --- | --- | --- | --- | --- | --- | --- |
| 2013 | **0.541**** | **0.542**** | **0.520**** | **0.452**** | **0.565**** | **0.635**** | **0.524**** |
| 2014 | **0.572**** | **0.497**** | **0.589**** | **0.317**** | **0.571**** | **0.632**** | **0.563**** |
| 2015 | **0.594**** | **0.608**** | **0.556**** | **0.289**** | **0.647**** | **0.623**** | **0.556**** |
| 2016 | **0.554**** | **0.559**** | **0.527**** | **0.367**** | **0.543**** | **0.585**** | **0.505**** |
| 2017 | **0.590**** | **0.553**** | **0.580**** | **0.327**** | **0.556**** | **0.639**** | **0.531**** |
| 2018 | **0.546**** | **0.485**** | **0.536**** | **0.322**** | **0.516**** | **0.566**** | **0.438**** |
| 2019 | **0.526**** | **0.501**** | **0.507**** | **0.227**** | **0.542**** | **0.538**** | **0.443**** |
| 2013–2019 | **0.600**** | **0.600**** | **0.590**** | **0.564**** | **0.630**** | **0.649**** | **0.622**** |

*Values in bold indicate statistical significance (*p<0.05, **p<0.001).*

Supplementary Table S6. Results of multivariable Bayesian models (Model 0 and Model 1) for age-standardized scrub typhus incidence in South Korea, 2013 to 2019, presented as coefficients (95% credible intervals).

|  | Spatial models^a^ | | Temporal models^b^ | | Spatiotemporal models^c^ | |
| --- | --- | --- | --- | --- | --- | --- |
|  | Model 0^d^ | Model 1^e^ | Model 0^d^ | Model 1^e^ | Model 0^d^ | Model 1^e^ |
| WAIC^f^ | 223,960.9 | 211,780.2 | 141,405.6 | 119,029.2 | 75,014.8 | 75,019.1 |
| Financial independence (%) | - | -0.485  (-0.553 to -0.418) | - | -0.768  (-0.782 to -0.755) | - | -0.310  (-0.455 to -0.165) |
| Forest area ($m^{2}$) | - | 0.619  (0.298–0.952) | - | 0.058  (0.046–0.070) | - | 0.156  (-0.107–0.419) |
| Dry field farming area ($m^{2}$) | - | -0.664  (-0.956 to -0.365) | - | -0.231  (-0.247 to -0.216) | - | 0.066  (-0.217–0.349) |
| Woman farmer population (N) | - | 1.584  (1.527–1.642) | - | 0.412  (0.399–0.425) | - | 0.178  (-0.009–0.364) |

^a^Model including spatial terms. ^b^Model including temporal terms. ^c^Model including spatial and temporal terms, interaction term of spatial and temporal structured terms. ^d^Base model including only intercept. ^e^Model including potential risk factors other than maximum rodent suitability. ^f^WAIC, Watanabe-Akaike information criterion.

Supplementary Table S7. Results of sensitivity analysis using multivariable Bayesian models with scrub typhus case counts as the outcome and log-transformed population as an offset in South Korea, 2013 to 2019, presented as coefficients (95% credible intervals).

|  | Spatial models^a^ | | | Temporal models^b^ | | | Spatiotemporal models^c^ | | |
| --- | --- | --- | --- | --- | --- | --- | --- | --- | --- |
|  | Model 0^d^ | Model 1^e^ | Model 2^f^ | Model 0^d^ | Model 1^e^ | Model 2^f^ | Model 0^d^ | Model 1^e^ | Model 2^f^ |
| WAIC^g^ | 173,911.1 | 172,462.0 | 172,463.0 | 191,184.9 | 145,722.3 | 139,867.6 | 88,075.6 | 88,053.4 | 88,045.0 |
| Maximum rodent suitability | - | - | -0.405  (-2.915–2.105) | - | - | 0.354  (0.344–0.364) | - | - | 0.225  (0.118–0.330) |
| Financial independence (%) | - | -0.030  (-0.072–0.013) | -0.030  (-0.072–0.013) | - | -0.620  (-0.629 to -0.612) | -0.592  (-0.601 to -0.584) | - | -0.254  (-0.317 to -0.190) | -0.258  (-0.323 to -0.193) |
| Forest area ($m^{2}$) | - | 1.853  (1.107–2.598) | 1.855  (1.109–2.60) | - | 0.211  (0.202–0.220) | 0.198  (0.188–0.207) | - | 0.267  (0.125–0.410) | 0.261  (0.121–0.403) |
| Dry field farming area ($m^{2}$) | - | 0.219  (-0.302–0.741) | 0.220  (-0.302–0.741) | - | -0.155  (-0.165 to -0.145) | -0.112  (-0.122 to -0.101) | - | 0.048  (-0.101–0.199) | 0.052  (-0.101–0.204) |
| Woman farmer population (N) | - | 0.786  (0.747–0.825) | 0.786  (0.747–0.825) | - | 0.327  (0.317–0.337) | 0.211  (0.201–0.221) | - | 0.064  (-0.012–0.141) | 0.041  (-0.036–0.118) |

^a^Model including spatial terms. ^b^Model including temporal terms. ^c^Model including spatial and temporal terms, interaction term of spatial and temporal structured terms. ^d^Base model including only intercept. ^e^Model including potential risk factors other than maximum rodent suitability. ^f^Model including potential risk factors including maximum rodent suitability. ^g^WAIC, Watanabe-Akaike information criterion.

Supplementary Table S8. Results of multivariable Bayesian spatiotemporal models for scrub typhus incidence stratified by gender in South Korea, 2013 to 2019, presented as coefficients (95% credible intervals).

|  | Men  (39,688, 41.5%) | Women  (55,913, 58.5%) |
| --- | --- | --- |
| WAIC^a^ | 51,667.2 | 61,039.1 |
| Maximum rodent suitability | 0.162 (0.066–0.259) | 0.207 (0.102–0.312) |
| Financial independence (%) | -0.277 (-0.340 to -0.213) | -0.274 (-0.339 to -0.208) |
| Forest area ($m^{2}$) | 0.302 (0.175–0.429) | 0.286 (0.147–0.426) |
| Dry field farming area ($m^{2}$) | 0.006 (-0.112–0.126) | 0.080 (-0.053–0.216) |

^a^WAIC, Watanabe-Akaike information criterion.

Supplementary Table S9. Results of sensitivity analysis using multivariable Bayesian spatiotemporal models with a neighbor matrix constructed using k-nearest neighborhood (k=5) in South Korea, 2013 to 2019, presented as coefficients (95% credible intervals).

|  | Total population^a^ | Men | Women | 0–39 years of age | 40–59 years of age | 60–79 years of age | ≥80 years of age |
| --- | --- | --- | --- | --- | --- | --- | --- |
| WAIC^b^ | - | 51,595.7 | 60,889.1 | 15,647.2 | 39,878.8 | 54,244.5 | 17,181.0 |
| Maximum rodent suitability | - | 0.159  (0.063–0.255) | 0.200  (0.095–0.305) | 0.018  (-0.081–0.116) | 0.143  (0.047–0.240) | 0.248  (0.152–0.341) | 0.092  (0.011–0.174) |
| Financial independence (%) | - | -0.279  (-0.342 to -0.214) | -0.262  (-0.328 to -0.195) | -0.349  (-0.431 to -0.267) | -0.260  (-0.327 to -0.192) | -0.213  (-0.277 to -0.148) | -0.133  (-0.196 to -0.070) |
| Forest area ($m^{2}$) | - | 0.294  (0.168–0.421) | 0.285  (0.146–0.424) | 0.474  (0.334–0.615) | 0.303  (0.175–0.432) | 0.159  (0.035–0.284) | 0.177  (0.083–0.270) |
| Dry field farming area ($m^{2}$) | - | 0.014  (-0.103–0.132) | 0.072  (-0.059–0.205) | 0.000  (-0.150–0.150) | -0.031  (-0.168–0.106) | 0.003  (-0.135–0.142) | -0.009  (-0.111–0.094) |
| Woman farmer population (N) | - | - | - | -0.105  (-0.212–0.004) | 0.038  (-0.045–0.122) | 0.066  (-0.013–0.146) | -0.070  (-0.156–0.016) |

^a^Model did not converge when including the total population using the k-nearest neighbor (k=5). Therefore, the result is not presented. ^b^WAIC, Watanabe-Akaike information criterion.
