# Supplementary material for: Distribution and Risk Factors of Scrub Typhus in South Korea, From 2013 to 2019: Bayesian Spatiotemporal Analysis
Source: JMIR Public Health Surveill. 2025 Sep 10;11:e68437. doi: 10.2196/68437 (PMC12422590; doi:10.2196/68437)
Supplement: Multimedia Appendix 2 [file publichealth-v11-e68437-s002.docx]

# Multimedia Appendix 2

## Methods

Supplementary methods. R code for the Bayesian hurdle Poisson spatiotemporal analysis

| library(INLA)  ###########################################  #Preparing data for modeling  ###########################################  #Split data for hurdle model  idx1 <- as.integer(df$age_std_inci_100000)==0  yy <- matrix(NA, ncol = 2, nrow = nrow(df))  #Making it as single data  yy[idx1, 1] <- as.integer(df$age_std_inci_100000)[idx1]  yy[!idx1, 2] <- as.integer(df$age_std_inci_100000)[!idx1]  #Prepare for spatial and temporal term  S <- length(unique(df$SGG)) #SGG refers to 250 municipal-level administrative boundaries of Korea  Temp <- length(unique(df$epiweek)) #epiweek refers to MMWR weeks  #Prepare data that INLA can read  btdf <- data.frame(inci=df$age_std_inci_100000,  idx=rep(1:S,Temp),  idx.int=rep(1:S,Temp),  temp=rep(1:Temp, each=S),  temp1=rep(1:Temp, each=S),  temp.int=rep(1:Temp, each=S),  new_rodent_p_max = scale(df$new_rodent_p_max),  finance_indep = scale(df$finance_indep),  forest=scale(df$forest),  dry_field=scale(df$dry_field),  female_farm_pop=scale(df$female_farm_pop)  )  ###########################################  #Modeling  ###########################################  #Spatial model0  fit01 <- inla(yy ~ 1+  f(idx, model = "bym", graph = adj, constr = TRUE), #adj, which is neighbor matrix, is constructed k-nearest neighborhood methods  data = btdf, family = c("binomial", "poisson"), control.compute = list(dic = TRUE, cpo = TRUE, waic=TRUE, return.marginals.predictor=TRUE))  #Temporal model0  fit02 <- inla(yy ~ 1+  f(temp, model = "ar1") + f(temp1, model="iid"),  data = btdf, family = c("binomial", "poisson"), control.compute = list(dic = TRUE, cpo = TRUE, waic=TRUE, return.marginals.predictor=TRUE))  #Spatiotemporal model0  fit03 <- inla(yy ~ 1+  f(idx, model = "bym", graph = adj, constr = TRUE)+  f(temp, model = "ar1") + f(temp1, model="iid")+  f(idx.int, model="besag", graph = adj, group=temp.int, control.group=list(model="ar1")),  data = btdf, family = c("binomial", "poisson"), control.compute = list(dic = TRUE,cpo = TRUE, waic=TRUE, return.marginals.predictor=TRUE))  #Spatial model1  fit11 <- inla(yy ~ 1+  f(idx, model = "bym", graph = adj, constr = TRUE)+  finance_indep+forest+dry_field+female_farm_pop,  data = btdf, family = c("binomial", "poisson"), control.compute = list(dic = TRUE,cpo = TRUE, waic=TRUE, return.marginals.predictor=TRUE))  #Temporal model1  fit12 <- inla(yy ~ 1+  f(temp, model = "ar1") + f(temp1, model="iid") +  finance_indep+forest+dry_field+female_farm_pop,  data = btdf, family = c("binomial", "poisson"), control.compute = list(dic = TRUE,cpo = TRUE, waic=TRUE, return.marginals.predictor=TRUE))  #Spatiotemporal model1  fit13 <- inla(yy ~ 1+  f(idx, model = "bym", graph = adj, constr = TRUE)+  f(temp, model = "ar1") + f(temp1, model="iid")+  f(idx.int, model="besag", graph = adj, group=temp.int, control.group=list(model="ar1"))+  finance_indep+forest+dry_field+female_farm_pop,  data = btdf, family = c("binomial", "poisson"), control.compute = list(dic = TRUE,cpo = TRUE, waic=TRUE, return.marginals.predictor=TRUE))  #Spatial model2  fit21 <- inla(yy ~ 1+  f(idx, model = "bym", graph = adj, constr = TRUE)+  new_rodent_p_max+finance_indep+forest+dry_field+female_farm_pop,  data = btdf, family = c("binomial", "poisson"), control.compute = list(dic = TRUE,cpo = TRUE, waic=TRUE, return.marginals.predictor=TRUE))  #Temporal model2  fit22 <- inla(yy ~ 1+  f(temp, model = "ar1") + f(temp1, model="iid") +  new_rodent_p_max+ finance_indep+forest+dry_field+female_farm_pop,  data = btdf, family = c("binomial", "poisson"), control.compute = list(dic = TRUE,cpo = TRUE, waic=TRUE, return.marginals.predictor=TRUE))  #Spatiotemporal model2  fit23 <- inla(yy ~ 1+  f(idx, model = "bym", graph = adj, constr = TRUE)+  f(temp, model = "ar1") + f(temp1, model="iid")+  f(idx.int, model="besag", graph = adj, group=temp.int, control.group=list(model="ar1"))+  new_rodent_p_max+ finance_indep+forest+dry_field+female_farm_pop,  data = btdf, family = c("binomial", "poisson"), control.compute = list(dic = TRUE,cpo = TRUE, waic=TRUE, return.marginals.predictor=TRUE)) |
| --- |
